# 3D Domain Swapping Dimerization of the Receiver Domain of Cytokinin Receptor CRE1 From *Arabidopsis thaliana* and *Medicago truncatula*

**DOI:** 10.3389/fpls.2021.756341

**Published:** 2021-09-24

**Authors:** Linh H. Tran, Anna Urbanowicz, Michał Jasiński, Mariusz Jaskolski, Milosz Ruszkowski

**Affiliations:** ^1^Institute of Bioorganic Chemistry, Polish Academy of Sciences, Poznań, Poland; ^2^Department of Biochemistry and Biotechnology, Poznan University of Life Sciences, Poznań, Poland; ^3^Department of Crystallography, Faculty of Chemistry, A. Mickiewicz University, Poznań, Poland

**Keywords:** signal transduction, cytokinin, phosphorelay, 3D domain swapping, structure, flavodoxin-like fold

## Abstract

Cytokinins are phytohormones regulating many biological processes that are vital to plants. CYTOKININ RESPONSE1 (CRE1), the main cytokinin receptor, has a modular architecture composed of a cytokinin-binding CHASE (Cyclases/Histidine kinases Associated Sensory Extracellular) domain, followed by a transmembrane fragment, an intracellular histidine kinase (HK) domain, and a receiver domain (REC). Perception of cytokinin signaling involves (i) a hormone molecule binding to the CHASE domain, (ii) CRE1 autophosphorylation at a conserved His residue in the HK domain, followed by a phosphorelay to (iii) a conserved Asp residue in the REC domain, (iv) a histidine-containing phosphotransfer protein (HPt), and (v) a response regulator (RR). This work focuses on the crystal structures of the REC domain of CRE1 from the model plant *Arabidopsis thaliana* and from the model legume *Medicago truncatula*. Both REC domains form tight 3D-domain-swapped dimers. Dimerization of the REC domain agrees with the quaternary assembly of the entire CRE1 but is incompatible with a model of its complex with HPt, suggesting that a considerable conformational change should occur to enable the signal transduction. Indeed, phosphorylation of the REC domain can change the HPt-binding properties of CRE1, as shown by functional studies.

## Introduction

Cytokinins are hormones that regulate plant growth and development (Wybouw and De Rybel, 2019). They affect *inter alia* embryogenesis, root and shoot meristem activity, vasculature and organ development. In legumes, cytokinins also play a central role in the modulation of nitrogen-fixing symbiosis (Miri et al., 2016). They are signaling molecules, conveying information from the symbiotic bacteria to the inner parts of the root, where cytokinins are required for cortical cell divisions/nodule formation (Reid et al., 2017). Moreover, cytokinins act as a negative regulator of infection within rhizodermis and, as a part of systemic autoregulatory mechanisms, control the number of nodules (Gamas et al., 2017). Since their discovery in 1955 during the quest for factors promoting cell division (Miller et al., 1955a,b), a lot of work has been invested to better understand cytokinins biosynthesis, molecular transport, metabolism (Kieber and Schaller, 2018), and signaling pathways (Steklov et al., 2013).

**Figure 1:**
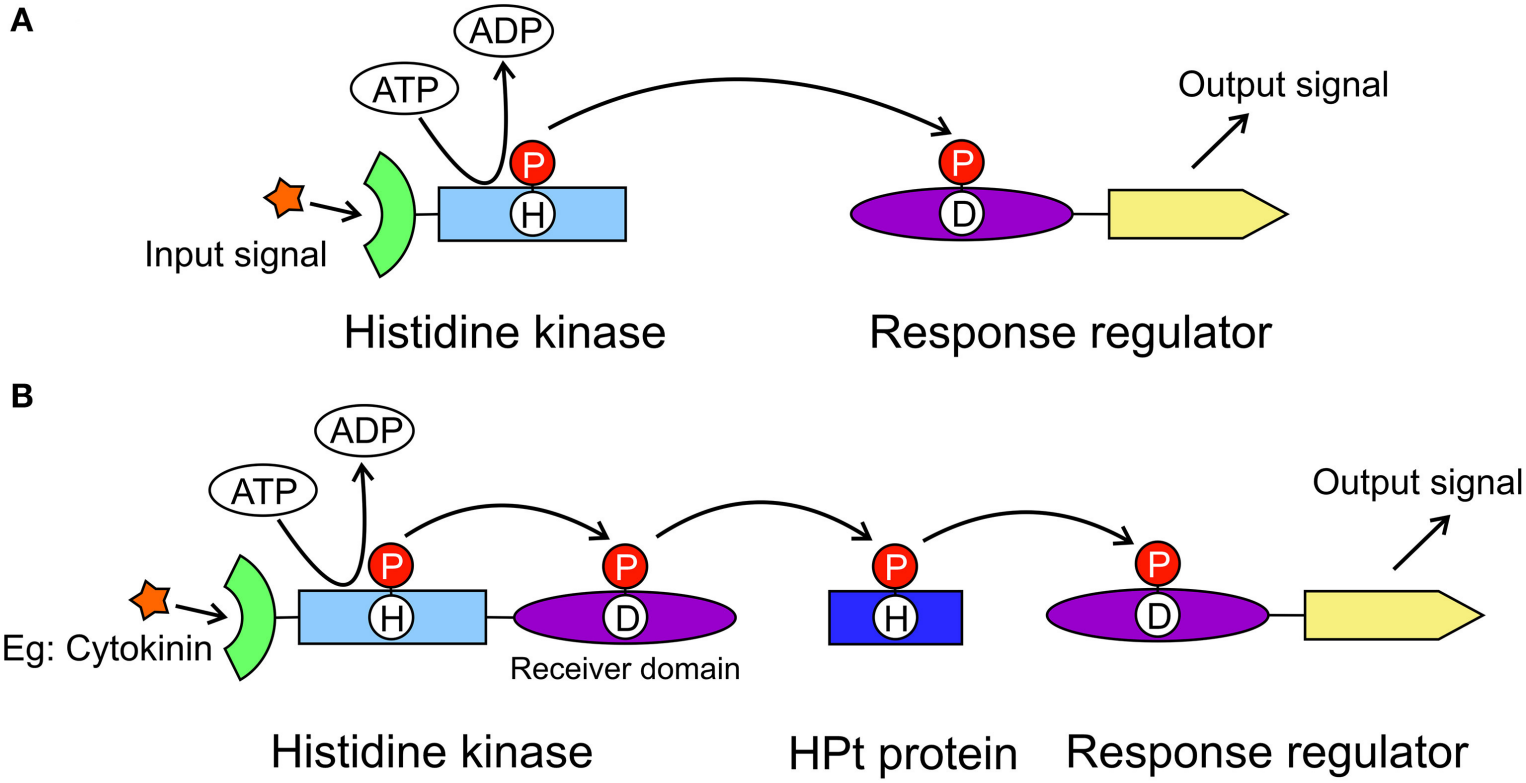
General models of the two-component system and the multistep phosphorelay system. The two-component system **(A)** is utilized in prokaryotes to transduce signals by a phosphorylation reaction. It includes a histidine kinase and a response regulator. The histidine kinase has an extracellular signal sensing domain (green) and a transmitter domain (light blue) where a conserved His residue will autophosphorylate. The phosphate group (red circle) is then transferred to the receiver domain (purple) of the response regulator resulting in the output signal from the effector domain (yellow). The multistep phosphorelay system **(B)** has evolved from the two-component system, in which the histidine kinase has an additional receiver domain. The multistep phosphorelay also has a histidine-containing phosphotransfer protein (dark blue) as the shuttle between the kinase and the response regulator.

The model for cytokinin perception and signaling involves a His-Asp multistep phosphorelay that is a more complex variant of the two-component system (TCS) utilized by prokaryotes (Figure 1A). The cytokinin transduction pathway comprises a histidine kinase (HK) receptor and the downstream elements histidine-containing phosphotransfer proteins (HPts) and response regulators (RRs, Figure 1B) (Sheen, 2002; Heyl and Schmülling, 2003; Kakimoto, 2003). In *Arabidopsis thaliana*, AHK4/CRE1/WOL (for *Arabidopsis* HISTIDINE KINASE4/CYTOKININ RESPONSE1/WOODEN LEG) was identified as a cytokinin receptor based on its sequence and its ability to bind cytokinins and to mediate activation of cytokinin responses (Mahonen et al., 2000; Inoue et al., 2001; Suzuki et al., 2001). Notably, in legumes, cytokinin signaling is specifically mediated by a receptor most closely related to AHK4/CRE1 of *A. thaliana* and referred to as MtCRE1 in *Medicago truncatula*, or histidine kinase 1 (LHK1) in *Lotus japonicus*. Both MtCRE1 and LHK1 are essential for the formation of nitrogen-fixing nodules on the root systems (Gonzalez-Rizzo et al., 2006; Murray et al., 2007). In *M. truncatula*, CRE1 is a positive regulator of nodulation but also acts as a negative regulator of lateral root formation and hence is crucial for root organogenesis (Gonzalez-Rizzo et al., 2006).

CRE1 has a modular architecture. It is made up of (N-to-C termini direction) an extracellular cytokinin-binding CHASE (Cyclases/Histidine kinases Associated Sensory Extracellular) domain, followed by a transmembrane fragment, an intracellular HK domain, and a receiver domain (REC). In plants, when a cytokinin molecule binds to the sensor domain, it triggers a phosphorylation cascade that starts with autophosphorylation of a conserved His residue in the kinase domain (Figure 1B). The phosphorylated His transfers the phosphate group to a conserved Asp in the REC domain. The second element, HPt, binds to the REC domain. The phosphoryl group is then transferred from Asp to the conserved His residue of HPt. Next, HPt dissociates from the REC domain and travels through the cytoplasm into the nucleus, where it phosphorylates the third element, a response regulator RR. There are two types of RR proteins, type A-RR and type B-RR. Type B-RRs trigger the transcription of certain genes, whereas type A-RRs act as feedback regulators of the cytokinin signal (Kakimoto, 2003).

In *A. thaliana*, there are six histidine kinases, of which three are cytokinin receptors (CRE1/AHK4, AHK2, AHK3), six AHP (*Arabidopsis* HPt) proteins, and over 20 RR proteins (Huo et al., 2020). The three cytokinin receptors show overlapping functions, and AHPs are not specific to particular AHKs (Huo et al., 2020), although they are expected to have different affinities. Similarly, each AHP can interact with more than one downstream RR (Huo et al., 2020). In *M. truncatula*, there are four HKs that have redundant expression patterns at early nodulation stages but diverge in differentiated nodules, although *MtCRE1* (sometimes referred to as *MtCHK1*) has dominant expression at all stages. Interestingly, *M. truncatula* has 10 genes encoding HPts, including two where the phosphorylable His are replaced with Asn and two with a His/Arg substitution (Tan et al., 2019). Moreover, *M. truncatula* contains 32 predicted RR proteins (Tan et al., 2019). It has been postulated that in *M. truncatula* different HKs/cytokinin signaling pathways regulate not only nodule initiation but also later developmental stages, and that legume-specific determinants encoded by the *MtCRE1* gene are required for nodulation stages later than initiation (Boivin et al., 2016). Interestingly, an *AHK4/CRE1* genomic locus from the aposymbiotic *A. thaliana* plant rescues nodule initiation but not nitrogen fixation in *M. truncatula cre1* defective mutants. To pinpoint the specificities of cytokinin perception and action, there is now a critical need not only to identify the downstream gene sets involved in the response but also to determine the molecular structures of all cascade components. The structures will provide hints about interactions and interrelations in this vital signaling pathway.

So far, several structures of different elements from the cytokinin signaling pathway have been solved. The structures of the sensor domain of the receptor in complexes with various cytokinins were presented by Hothorn et al. (2011) (PDB ID: 3T4J, 3T4K, 3T4L, 3T4O, 3T4Q, 3T4S, 3T4T). As regards the HPt proteins, a number of structures have been published, including MtHPt1 (3US6) (Ruszkowski et al., 2013) and MtHPt2 (4G78), ZmHP2 (1WN0) (Sugawara et al., 2005), OsHP1 (2Q4F) (Levin et al., 2007), AHP2 (4PAC) and AHP1 (4EUK). Structures of some REC domains of related but cytokinin-independent proteins were also solved, including ETR1 (PDB ID: 1DCF) (Muller-Dieckmann et al., 1999), CKI1 (3MM4) (Pekarova et al., 2011) and AHK5 (4EUK) (Bauer et al., 2013), but they all showed a monomeric structure. In this work, we focus on the crystal structures of the REC domain of the CRE1 receptor proteins from *M. truncatula*, and *A. thaliana*. As the CRE1 proteins are dimers, this work provides an important first glimpse into the dimeric REC structure. Both presented CRE1-REC domains dimerize through 3D domain swapping, suggesting that a considerable change of conformation must occur upon the phosphorylation reaction to allow HPt binding.

## Results and Discussion

### The Overall Structure of the CRE1-REC Domain

**Figure 2:**
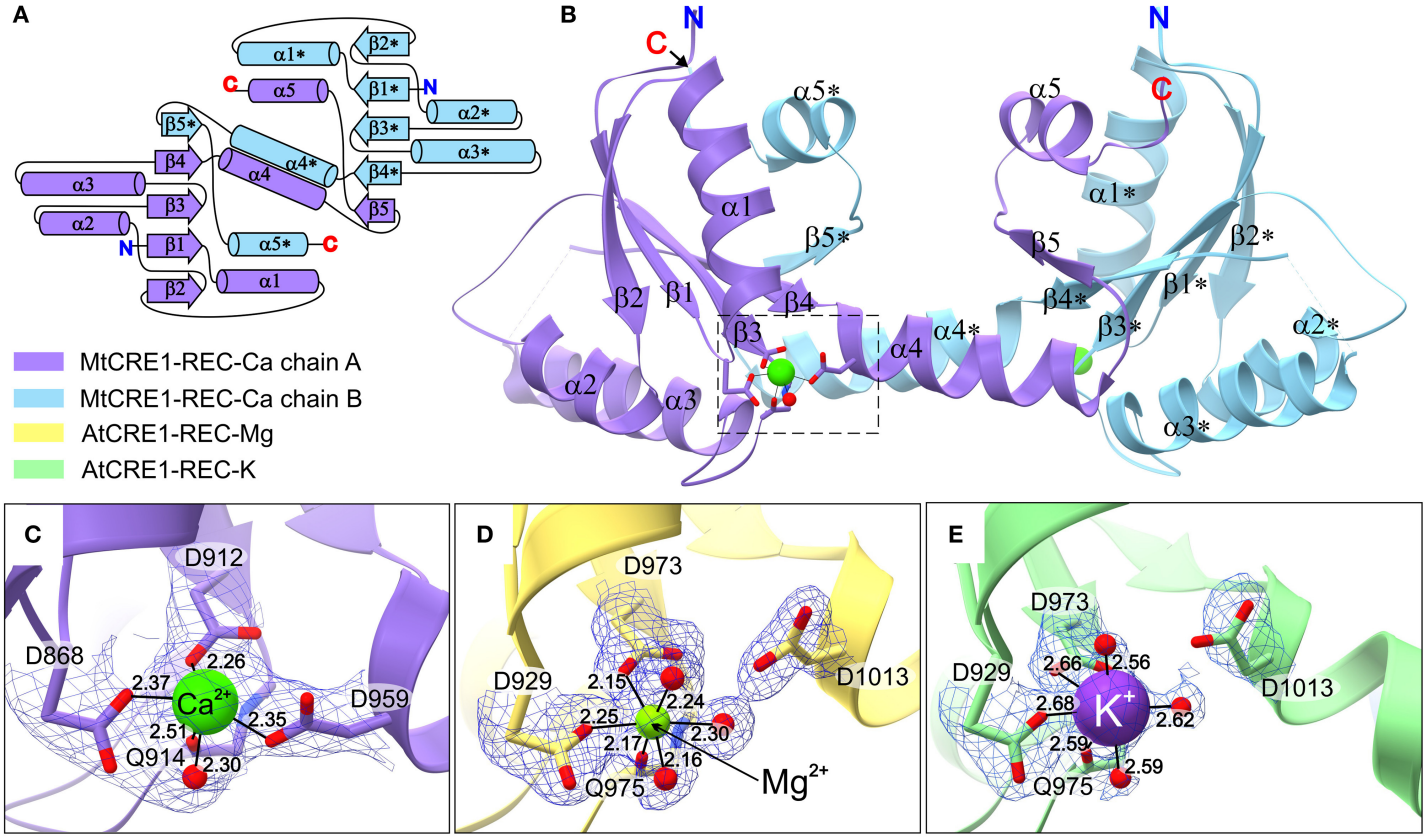
The structure of CRE1-REC. **(A)** Shows the topology diagram, while **(B)** illustrates the quaternary structure (in cartoon representation) of MtCRE1-REC. The homodimer consists of two subunits, chain A (purple) and chain B (blue). Each chain has a flavodoxin-like core (β1 → β4), in which a parallel β-sheet (β2−β1−β3−β4) is flanked by two layers of helices (α1−α5* and α2−α3; an asterisk denotes an element of the other subunit) and a 3D swapping subdomain (α4−β5−α5). In this structure, each subunit binds a Ca^2+^ cation **(C)**. AtCRE1-REC has a similar structure with two species of the metal cation, Mg^2+^ (yellow) or K^+^ (green), shown in **(D,E)**, respectively. The metal coordination is shown in 2mFo-DFc electron density contoured at the 1σ level, and the coordination bonds are marked as thin black lines, with bond distances in Å.

The crystal structure of MtCRE1-REC (Figures 2A–C) was solved and refined using X-ray diffraction data extending to 2.5 Å resolution (Table 1). MtCRE1-REC, residues Ser856-Phe994, crystallized in space group *P*3_1_21 with one protein subunit in the asymmetric unit, forming a crystallographic dimer with its twofold-symmetric copy. The PDBePISA server (Krissinel and Henrick, 2007) predicts the existence of a homodimer in solution. Overall, the refined model includes 126 residues, three water molecules, and one calcium cation coordinated by the protein. There is one missing fragment of 13 residues (936–948) in the electron density map.

**Table 1:** Diffraction data and refinement statistics.

**Data collection**	**AtCRE1-REC-K**			**AtCRE1-REC-Mg**	**MtCRE1-REC-Ca**		
Beamline	P13 beamline of the PETRA III storage ring at DESY Hamburg						
Wavelength (Å)	1.0000			0.9763	0.9755		
Temperature (K)				100			
Space group	*P*2_1_2_1_2			*P*2_1_2_1_2	*P*3_1_21		
Unit cell parameters a/b/c (Å)	90.3/101.4/33.6			88.2/99.2/35.6	60.2/60.2/80.4		
	**Overall** ^**a**^	**Inner** ^**a**^	**Outer** ^**a**^		**Overall** ^**a**^	**Inner** ^**a**^	**Outer** ^**a**^
Resolution (Å)	41.25–2.15	41.25–7.16	2.39–2.15	43.23–1.70 (1.80–1.70)^**b**^	52.12–2.50	52.12–7.20	2.89–2.50
Unique reflections	10,946	546	544	35,267	3,859	298	296
Multiplicity	12.9	11.6	11.5	12.9 (13.2)^**b**^	6.2	5.3	6.0
Ellipsoidal completeness (%)	92.8	99.8	63.9		88.7	99.7	59.8
Spherical completeness (%)	62.2	99.8	11.4	99.9 (99.8)^**b**^	62.6	99.7	13.8
Rmerge (%)	17.6	6.6	123.0		9.7	3.8	102.8
< I/σ(I)>	11.4	28.7	2.6	22.9 (1.56)^**b**^	12.9	32.9	2.0
CC(1/2)	0.99	0.99	0.73	0.99	0.99	0.99	0.78
**Refinement**							
R_free_ reflections	554			1058	191		
**No. of non-H atoms**							
Protein	1,968			2,076	992		
Metals	2			2	1		
Solvent (water/other)	41/0			182/12	3/0		
Rwork/Rfree (%)	20.90/28.40			19.40/24.60	17.30/22.10		
**RMSD from ideal geometry**							
Bond lengths (Å)	0.01			0.01	0.01		
Bond angles (°)	1.01			1.07	1.13		
**Ramachandran statistics (%)**							
Favored/allowed/outliers	97.99/2.01/0.00			98.09/1.91/0.00	92.62/6.56/0.82		
PDB ID	7P8C			7P8D	7P8E		

a *Data processing statistics are given separately for: all reflections (left column), inner shell (middle column), and outer shell (right column)*.

b *Values in parentheses are for the last resolution shell*.

The structure of AtCRE1-REC was determined in two isomorphous variants of space group *P*2_1_2_1_2 with two protein subunits (chains A and B) in the asymmetric unit forming a homodimer. Homodimerization of AtCRE1-REC in solution was verified by the comparative analysis with different proteins using native polyacrylamide gel electrophoresis (Native-PAGE, Supplementary Figure 1). The structure of the AtCRE1-REC-Mg^2+^ complex (referred to as AtCRE1-REC-Mg), determined at 1.7 Å resolution, encompasses residues Leu918-Ser1057, extended at the N-terminus by a Ser-Asn-Ala cloning artifact. In the final model, subunit A is comprised of 139 residues and subunit B of 127 residues. In chains A and B, there are two, and 15 missing residues (Ser1056-Ser1057/Ser1043-Ser1057), respectively, which could not be modeled in the electron density because of disorder. Each subunit binds a magnesium cation. There are 182 water molecules, one ethylene glycol and one (4S)-2-methyl-2,4-pentadiol (MPD) molecule in the asymmetric unit.

The second structure of AtCRE1-REC (referred as AtCRE1-REC-K), determined at 2.15 Å resolution, corresponds to a construct with a 9-residue C-terminal truncation and has potassium instead of magnesium in the metal binding site. Its chain A is comprised of 134 residues, including Ala917 of the cloning linker, and chain B of 120 residues, including two residues of the linker (Asn916–Ala917). While chain A has the complete sequence (Leu918–Phe1048), chain B lacks 13 residues at the C-terminus (Glu1036–Phe1048). The structure contains 41 water molecules. In this paper, the AtCRE1-REC-K structure is mainly used for comparative analysis of the metal binding site. For protein structure analysis, the full C-terminal construct from the Mg^2+^ complex above is used.

It is noted that the AtCRE1-REC (both isomorphous variants) and MtCRE1-REC proteins were obtained in different ways. For AtCRE1-REC, the coding sequence of only the receiver domain was cloned into a plasmid and expressed. The protein was then purified and subjected to crystallization. For MtCRE1-REC, the coding sequence of the full intracellular domain (MtCRE1-IC) was cloned and expressed, followed by limited proteolysis with thermolysin. The intracellular domain was digested, leaving only the REC domain that was used for crystallization.

Both MtCRE1-REC and AtCRE1-REC belong to the α/β folding class and their topology pattern includes five α/β motifs (Figure 2A). They possess the same flavodoxin-like structure, in which a four-stranded parallel β-sheet (β1–β4) is flanked on both sides by two layers of α helices (α1–α5^*^ and α2–α3; an asterisk denotes an element of the other subunit). In MtCRE1-REC, the α4 helix and its following elements (Ala958–Phe994) jut toward the complementary protein molecule in the dimer, where the β5 strand joins the four-stranded parallel β sheet of the second subunit (Figures 2A,B). Herein, the β1-α1-β2-α2-β3-α3-β4 fragment is referred to as the N-subdomain and the α4-β5-α5 fragment as the C-subdomain or the swapping subdomain; Thr957 (Thr1011 in AtCRE1) constitutes the subdomain boundaries (Bennett et al., 1995; Jaskolski, 2001, 2013). This mutual 3D domain swapping act is a unique feature of CRE1-REC compared to other published structures of REC domains from plants, including AHK5 (Bauer et al., 2013), as discussed in detail later.

### The Dimeric CRE1-REC Is Remarkably Different From Monomeric AHK5-REC

**Figure 3:**
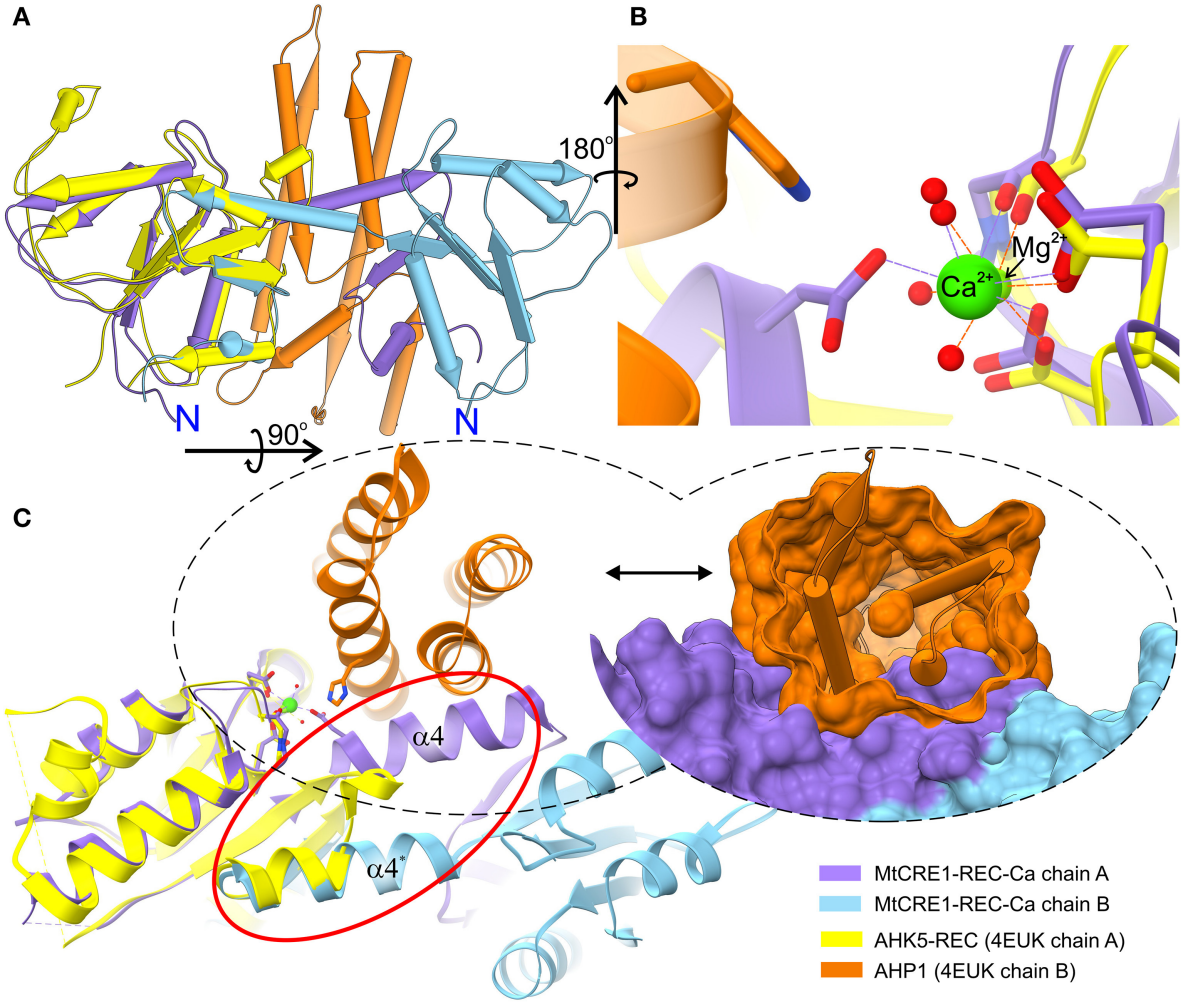
Superposition of the MtCRE1-REC dimer (purple/blue) onto the AHK5-REC (yellow) complex with AHP1 (orange) **(A)**. MtCRE1-REC and AHK5-REC share a similar flavodoxin-like core structure. They also have a similar metal-binding site, shown in stick representation **(B)**. While MtCRE1-REC binds Ca^2+^, AHK5-REC binds Mg^2+^. An attempt to superpose the two structures leads to severe clashes between AHP1 (orange surface) and MtCRE1-REC (purple surface), as shown in **(C)**. The clashes occur because of the presence of the swapping domain (circled in red). In MtCRE1-REC, the purple α helix juts toward the second subunit (blue), while in AHK5-REC it flips back in the opposite direction (yellow).

Compared to the receiver domain of AHK5 (PDB ID 4EUK), the cores of both MtCRE1-REC and AtCRE1-REC are rather similar, except for a key quaternary structure difference, namely that in contrast to the dimeric structure of our CRE1-REC domains, AHK5 is monomeric and does not have the distinct swapping subdomain. In spite of the fact that the two sequences (AHK5-REC and MtCRE1-REC) share only 34% identity (Supplementary Figure 2), the two structures superpose very well, as shown in Figure 3A (RMSD of 0.88 Å). In the 4EUK structure, AHK5-REC coordinates a magnesium ion (Figure 3B). The coordination sphere is formed by six oxygen atoms, including two Oδ atoms of Asp785 and Asp828, one backbone O atom of Cys830, and three water molecules. Instead of protruding outward to interact with the complementary subunit, the α5 helix of AHK5-REC flips backward (red ellipse in Figure 3C) and completes the fold of “its own” subunit, where the β5 strand joins the β-sheet of the same molecule. Interestingly, the α5 helix is well-superposed on the α5 helix of the second molecule in the MtCRE1-REC structure. In the 4EUK structure, AHK5-REC binds its cognate AHP1 partner so that the catalytic His79 of AHP1 points toward the metal binding site of AHK5-REC. The catalytic Asp828 of AHK5-REC, the Mg^2+^ ion, and the reactive His79 of AHP1 are almost in-line (Bauer et al., 2013). However, such binding would be impossible in the MtCRE1-REC structure due to the fundamental difference in the quaternary structure. As shown in Figure 3C, the AHP1 (orange surface) in this arrangement would have huge steric clashes with the α4 helix of MtCRE1-REC; the same applies to AtCRE1-REC (not shown). Thus, the 3D domain swapping feature would prevent the cognate HPt from binding CRE1.

### The Metal Cation at the Phosphorylation Site Influences the Conformation of the Swapped Region, Suggesting That Phosphorylation Triggers a More Significant Structural Change

**Figure 4:**
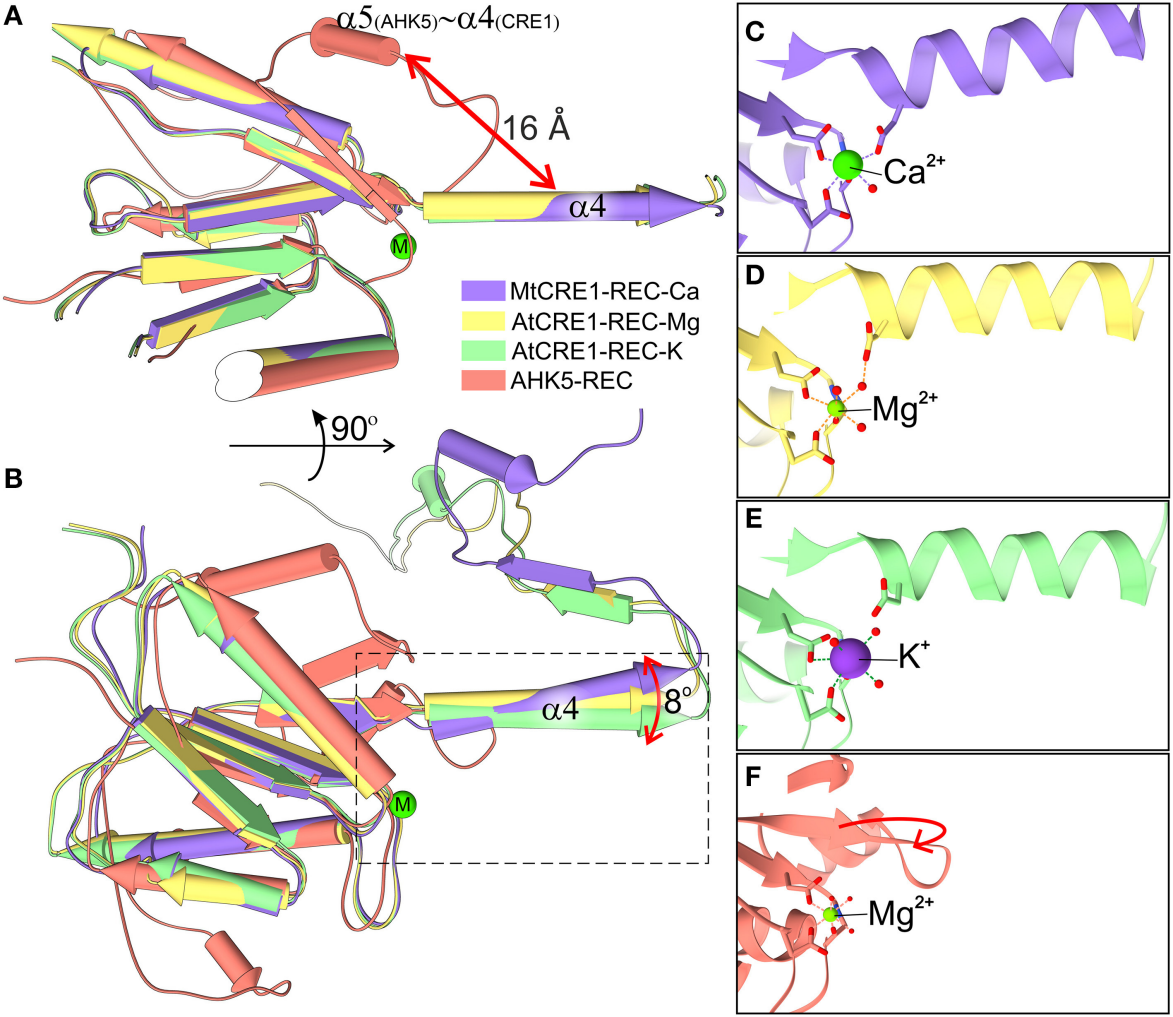
Superposition of four structures of the REC domain (MtCRE1-REC-K in purple, AtCRE1-REC-Mg in yellow, AtCRE1-REC-K in green, AHK5-REC in salmon). They are shown in pipe (α helix) and plank (β strand) representation in two views **(A,B)** emphasizing the different orientation of helix α4 in the domain-swapped and unswapped states and **(B)** the variable orientation of helix α4 in the domain swapped structures with different metal cations. The active site is shown in stick representation for MtCRE1-REC-Ca **(C)**, AtCRE1-REC-Mg **(D)**, AtCRE1-REC-K **(E)** and AHK5-REC **(F)**, where the red arrow indicates the helix unswapping direction.

The three presented structures of CRE1-REC contain different metal cations bound at equivalent metal binding sites. It is noted that AtCRE1-REC and MtCRE1-REC share 78% sequence identity (Supplementary Figure 2) and that their structures superpose well (Figure 4, RMSD of 0.56 Å for the core N-subdomain β1 → β4); thus, the difference in the metal binding site arises from the nature of the metal. In the MtCRE1-REC structure, the coordination sphere of the calcium ion comprises five oxygen atoms, three Oδ atoms from Asp868, Asp912, Asp959, one backbone O from Gln914, and one water molecule (Figure 2C). The lengths of the Ca-O coordination bonds range from 2.26 Å to 2.51 Å, in agreement with the analysis by Yang et al. (2002). In the AtCRE1-REC-Mg complex, the octahedral coordination sphere consists of six oxygen atoms: two Oδ of Asp929 and Asp973, one backbone O atom of Gln975 and three water molecules (Figure 2D). The lengths of the Mg-O coordination bonds range from 2.15 to 2.30 Å. A similar octahedral coordination sphere of the same six oxygen atoms is found in the potassium complex of AtCRE1-REC (Figure 2E). The K-O coordination bond lengths range from 2.56 to 2.68 Å.

The Oδ atom of Asp959 is directly involved in the coordination sphere in the MtCRE1-REC-Ca structure, but its equivalent (Asp1013) in the AtCRE1-REC structures is not. It can be speculated that the radius and charge of the metal cation cause this difference. Among the three metal cations, Mg^2+^ ion is the smallest divalent cation, resulting in the highest local charge. The Metal-O bond between Mg^2+^ and Oδ of Asp929 is shorter than the bond between Ca^2+^ and Oδ of the corresponding Asp868 (2.25 and 2.37 Å, respectively). As a result, the Mg^2+^ ion lies farther away from Asp1013 and a water molecule fills up the coordination sphere, bridging it with the Oδ atom of Asp1013 (helix α4). On the other hand, the Ca^2+^ ion in the MtCRE1-REC-Ca structure has a larger radius, and with strong electrostatic attraction, it pulls the Oδ atom of Asp959 (helix α4) into the coordination sphere. However, even though the potassium cation in the AtCRE1-REC-K structure has the largest radius among the three metal cations, its charge is only +1, which apparently is not sufficient to pull Asp1013 into the coordination sphere. Instead, there is a water molecule replacing the interaction of the Oδ atom of Asp1013. Even though this is apparently only a minor change, it has a direct influence on the orientation of the α4 helix, as shown in Figure 4B. In MtCRE1-REC-Ca, the N-end of the α4 helix is pulled toward the metal binding site, with a lever effect at the C-end of the helix, which moves farther away compared to AtCRE1-REC (Figures 4C vs. D,E).

Since a change of electrostatic charge from +2 to +1 can already cause clearly visible structural rearrangements, one may expect that more significant changes will occur upon phosphorylation. Once the Asp912 residue in MtCRE1 (Asp973 in AtCRE1) is phosphorylated, it bears a huge negative charge, leading to strong repulsion of the negatively charged Asp959 in the α4 helix of MtCRE1 (Asp1013 in AtCRE1) and triggering a conformational transition.

Indeed, a similar situation was observed in bacterial sporulation regulator Spo0A (Supplementary Figures 3, 4) despite the swapping element being the α5 helix counterpart, where there is a noticeable deviation of the N-end of the α4 helix between the unphosphorylated and the phosphorylated state (Supplementary Figure 4). The Spo0A protein provides an example of quaternary structure changes upon phosphorylation. Its receiver domain also acquires a flavodoxin-like fold. The Spo0A receiver domain exists in monomeric phosphorylated state (Lewis et al., 1999) and dimeric unphosphorylated state (Lewis et al., 2000). The dimer is formed by 3D domain swapping of the last α5 helix (corresponding to α5 in CRE1-REC structures) in a structural transition caused by a *cis-trans* isomerization of the Lys106-Pro107 peptide, which is located in a loop. The similarity of the domain swapping mechanism is in contrast to the low sequence identity of 22% between MtCRE1-REC and Spo0A (Supplementary Figure 3), suggesting that phosphorylation-triggered dimer-to-monomer transition has evolved very early.

### An Unswapping Event Most Likely Occurs Prior to HPt Binding

From a comparison with the AHK5/AHP1 complex, it is evident that there must be a conformational change that would enable CRE1-REC interaction with its cognate HPt. A possibility that HPt binds CRE1-REC at a different binding site compared to the AHK5/AHP1 complex is highly unlikely in light of the fact that the phosphotransfer reaction requires a metal cation (typically Mg^2+^), which is a good marker of the area of interaction. In addition, the HPt acceptor His residue cannot be too far from the conserved Asp of the REC domain.

**Figure 5:**
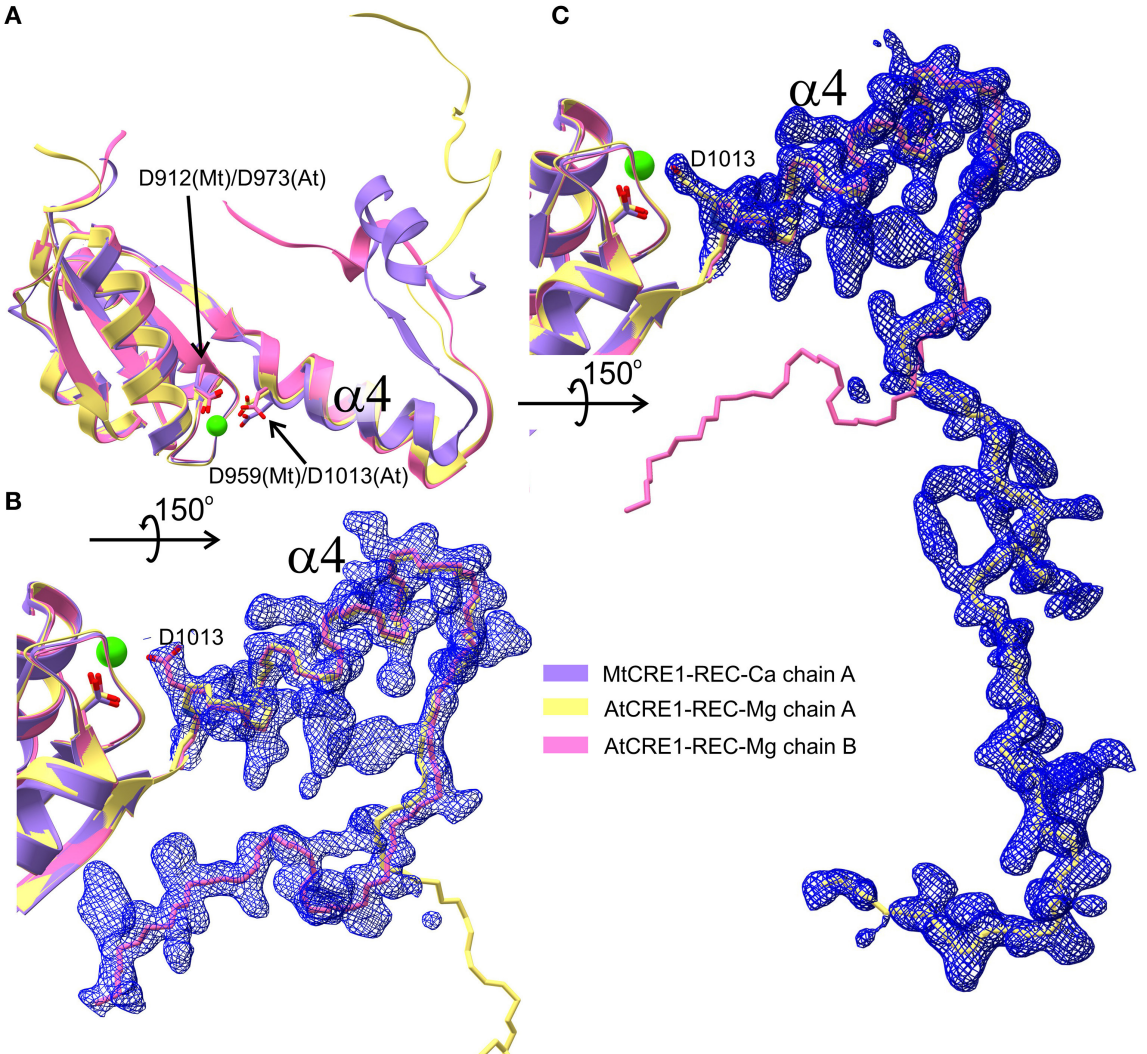
Three different conformations of the CRE1-REC swapping domain are superposed in **(A)** (cartoon representation), as found in MtCRE1-REC (purple), AtCRE1-REC chain A (yellow) and AtCRE1-REC chain B (pink). The open C-terminus of the AtCRE1-REC structure and its 2mFo-DFc electron density map contoured at the 1σ level is shown for subunit A **(C)** and subunit B **(B)** with the Cα traces in yellow and pink, respectively. The clear electron density fully supports the postulate of conformational change events taking place along the 3D domain swapping pathway.

As observed in several 3D swapping domains, e.g., in the other flavodoxin-like protein mentioned above, the core (N-subdomain) of the 3D swapped form resembles the monomeric fold (Bennett et al., 1995). In a 3D domain-swapped dimer, the swapping domain intertwines with an identical protein chain to recreate an interface as in the monomeric assembly (Bennett et al., 1995). Thus, it is most likely that the monomeric form of CRE1-REC might resemble its AHK5 homolog (Figure 4F). It means that the swapping subdomain would have to partially unfold in the linker region and reassemble with its own protein chain. It is of note that intrinsic structural flexibility of the swapping subdomain is observed in our AtCRE1-REC structures. In both chains there is no regular secondary structure in this region but nevertheless the electron density is very clear, as shown in Figure 5. In order to form a monomer, the α4 helix has to flip backward. As discussed above, a minor change in the metal cation site leads to a substantial change in the conformation of the α4 helix. When Asp912 (in MtCRE1 or Asp973 in AtCRE1) is phosphorylated, the site becomes negatively charged. This should cause a strong repulsion between Asp912-PO_4_ and Asp959 (in helix α4 of MtCRE1-REC or Asp973-PO_4_ and Asp1013 in AtCRE1) to trigger the conformation change.

As observed in Spo0A, the phosphorylation causes a *trans*-*cis* isomerization of a peptide bond, leading to the conformation change. As a result, the Lys106 side chain flips by 180° and is pulled over 14 Å into the coordination sphere of the phosphate group (Supplementary Figure 4) (Lewis et al., 2000). Another 3D domain swapping example is the CheY protein. CheY, the archaeal chemotaxis-linked protein, is the response regulator in the TCS which receives the phosphate group from CheA protein. Unphosphorylated and phosphorylated forms of CheY have been characterized (Quax et al., 2018), revealing that in both states it is a monomeric protein. However, recently, Paithankar et al. (2019) have described a dimeric form of the CheY protein. It has an open conformation, in which the N-subdomain (β1-α1-β2-α2-β3) of each subunit interacts with the C-subdomain (α3-β4-α4-β5-α5) of the other subunit. In this case, it has been suggested that the dimer formation is the result of cold-induced destabilization and high protein concentration in the crystallization experiments but is not necessarily related to physiological function.

**Figure 6:**
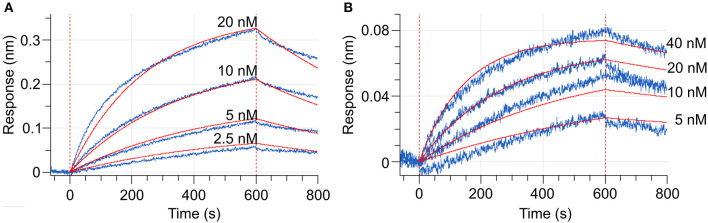
Biolayer interferometry (BLI) curves (baseline, association, dissociation, marked by vertical red dashed lines). **(A)** Shows the interaction between MtCRE1-IC and MtHPt1 in the presence of 2 mM MgCl_2_ and 0.5 mM ATP. The corresponding K_d_ value is 12 ± 0.06 nM. In the measurement, the MtHPt1 protein with N-terminal His-tag and MBP fusion was immobilized on Ni-NTA sensor at a concentration of 104 nM (6.3 μg/mL). The four MtCRE1-IC concentrations used in each BLI experiment were 2.5, 5, 10 and 20 nM. Each measurement included a parallel reference in which the sensors were immersed in the BLI buffer only to monitor and subtract unspecific ligand binding effect to the sensor. **(B)** Illustrates BLI curves for the interaction between MtCRE1-IC and MtHPt1 without ATP and MgCl_2_. The signal level is significantly lower despite the higher concentration of analyte.

To measure the changes in the MtCRE1-REC interaction with MtHPt1 that occur upon phosphorylation, we used biolayer interferometry (BLI). The binding curves were monitored and recorded in the presence and in the absence of 0.5 mM ATP and 2 mM MgCl_2_. The BLI response signal in the presence of ATP and MgCl_2_ was strong and allowed to calculate the *K*_d_ (Figure 6A), while the response signal in the absence of ATP and MgCl_2_ was too low to obtain binding parameters meeting standard quality criteria (Figure 6B). The *K*_d_ values estimated in three measurements (each involving four ligand serial dilutions) were in the range of 12–54 nM. The slight differences of K_*d*_ values among the measurements result from the fact that each time the protein preparations were purified anew. The BLI-estimated *K*_d_ values are much lower than the *K*_d_ value reported previously (14 μM) using the microscale thermophoresis (MST) technique (Ruszkowski et al., 2013), suggesting that labeling the protein for MST partially disturbed the interaction (Bierwagen et al., 2021). The minor signal in the absence of ATP and MgCl_2_ is likely attributable to a small fraction of CRE1 with unswapped receiver domain. Two hypotheses can be postulated to explain this small signal. The first possibility is that these two conformational states, swapped and unswapped, are in equilibrium, and the phosphorylation/dephosphorylation reaction of the receiver domain shifts this equilibrium. The second hypothesis is that a small fraction of CRE1 remained phosphorylated from recombinant production in *E. coli* and thus gave the minor signal. Nevertheless, the huge difference in the BLI signal in the presence and absence of ATP implies different binding between MtCRE1-IC and HPt1, which strongly supports the hypothesis of a phosphorylation-induced conformational change.

To gain further insights into which fragments of MtCRE1 are most affected by phosphorylation, we purified and incubated MtCRE1-IC with 0.5 mM of ATP and 2 mM of MgCl_2_ for one day, followed by limited proteolysis using thermolysin (0.5 μg/mL). The reference (incubation without ATP) and the sample were run on SDS-PAGE (Supplementary Figure 5) and the cut-out bands were analyzed by mass spectrometry (MS). The MS result (Supplementary Figure 6, Supplementary File 1) shows a difference in the digestion pattern. The fragment Thr933-Lys970, which corresponds to the α4 swapping helix (959–970) and the β4 strand (953–956), is only observed in the reference (ATP absent). This result is consistent with the phosphorylation-induced conformational change. In the reference, the α4 swapping helix is tightly bound to its dimer partner, protecting it from digestion by thermolysin. On the other hand, ATP causes a change in the conformation that makes the swapping element (α4) exposed and digested.

**Figure 7:**
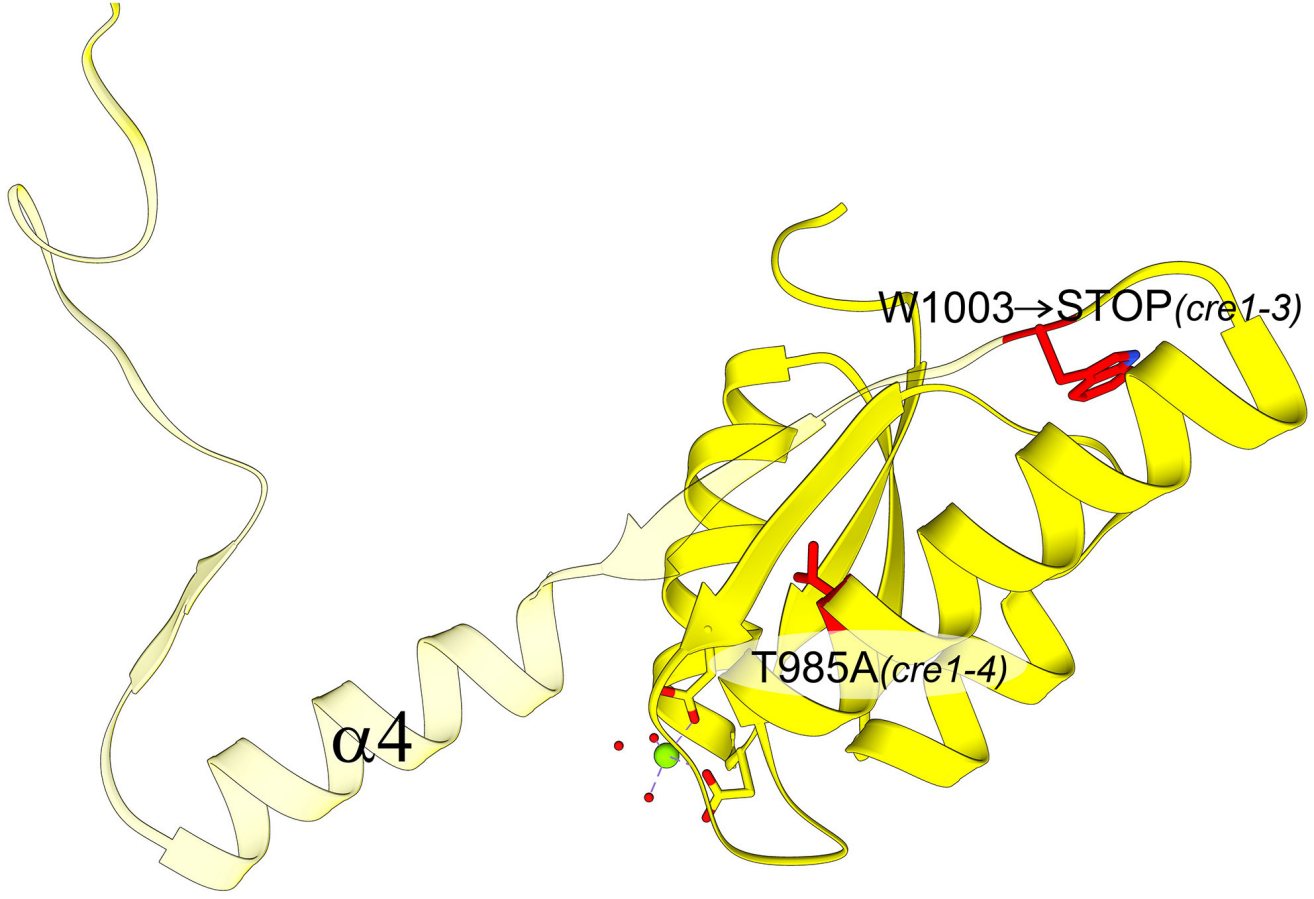
AtCRE1-REC with two mutation variants (residues marked in red): *cre1-3* and *cre1-4*. The view is rotated 180° around the y-axis with respect to Figure 5A. The *cre1-4* variant has a single amino acid substitution while the *cre1-3* mutation leads to a stop codon at Trp1003. The protein product of *cre1-3* is truncated (transparent fragment), losing its 3D swapping subdomain and displaying abnormal properties.

Our hypothesis about the importance of the swapping element is also consistent with the results of a genetic screen conducted on *A. thaliana* that was aimed at identifying genes involved in mediating the cytokinin effect during phosphate starvation response. The screen revealed several *cre1/ahk4* mutant alleles; all but one allele showed point mutations in either the HK domain or the REC domain (Franco-Zorrilla et al., 2002). While these mutations are informative about functionally relevant sites within CRE1, crucial for cytokinin-induced repression of phosphate starvation responses in *A. thaliana*, our structural data illuminate the molecular background behind some of these mutations. The *cre1-3* and *cre1-4* mutants within the AtCRE1-REC domain showed reduced sensitivity to cytokinin in the repression of phosphate starvation response (Franco-Zorrilla et al., 2002). While the *cre1-4* allele only has a T985A substitution, the *cre1-3* allele, which causes a more significant reduction in cytokinin sensitivity, has a mutation into a stop codon (Trp1003 → STOP Figure 7). Trp1003 lies in the loop between α3 and β4, just prior to the swapping domain (α4-β5-α5). Hence, this *cre1-3* mutation leads to a truncated protein without the swapping domain, explaining the severe reduction in cytokinin sensitivity mentioned in Franco-Zorrilla et al. (2002). It is uncertain whether the remaining activity can be attributed to the *cre1-3* protein product or perhaps to AHK2 and/or AHK3 taking over the signaling. Nonetheless, by mapping the mutations on the AtCRE1-REC protein structure, it becomes evident why cytokinin response in the *cre1-3* mutant is significantly more impaired compared to *cre1-4* (Franco-Zorrilla et al., 2002).

## Conclusions and Outlook

We have reported the structures of the dimeric form of the receiver domain of cytokinin receptor CRE1 from *A. thaliana* and *M. truncatula*. This dimeric form is very likely related with the dimerization of the receptor. Using for comparison a complex of its homolog, namely AHK5 with the downstream AHP1, we conclude that the CRE1-REC dimer must drastically change its conformation so that its HPt partner can bind. Moreover, different metal cations lead to different orientations of the swapping helix. Thus, we hypothesize that the driving force of this conformation change is the phosphorylation event. It is consistent with the kinetic assay, where we observed efficient binding between MtCRE1-IC and MtHPt1 only in the presence of ATP, *i.e.*, when phosphorylation was occurring. MS experiments further showed that phosphorylation changes the proteolysis pattern within the swapping element.

Structural data concerning CRE1 are still scarce, despite the fact that this protein plays a crucial role in cytokinin signal transduction. While structures of the cytokinin-binding CRE1-CHASE domain have been presented (Hothorn et al., 2011), and this work provides the first glimpse of the CRE1-REC domain, most of the protein structure, including the HK domain, remains unknown. Unfortunately, CRE1-IC and the full-length CRE1 are difficult to crystalize, possibly due to conformational heterogeneity and transmembrane regions, respectively. Currently, two approaches seem most viable (i) the divide-and-conquer approach, an example of which is provided in this paper, or (ii) the design of protein fusion constructs suitable for cryoelectron microscopy studies. That as well as other structural work is needed to fully elucidate the functionality of HK, HPt, and RR variants, and to explain their redundancy in some but not all plant responses in cytokinin signaling (Boivin et al., 2016). Finally, structural data enable better understanding of phenotypes revealed upon genetic screens dedicated to elucidation of cytokinin signaling pathway.

## Materials and Methods

### Cloning, Overexpression and Purification of the Proteins

For MtCRE1-IC (Uniprot ID: G7LCC3, GenBank: XM_003630966.4), the intracellular domain (Ile352-Ser1003) was produced as described previously (Ruszkowski et al., 2013). Thermolysin (Hampton Research) protease was added to the protein sample after size-exclusion chromatography (SEC) at the final concentration of 0.5 μg/mL. The next day, the sample was purified using a Superdex 75 16/60 column and used for crystallization.

The sequence coding for AtCRE1-REC (Uniprot ID: Q9C5U0-2, GenBank: AB049934.1) was taken from UniProt, while the REC domain boundaries were determined based on the MtCRE1-REC structure. Only the receiver domain was amplified by PCR reaction using KOD Hot Start Master Mix polymerase (Merck) and *A. thaliana* cDNA as the template. Two variants were made, a complete receiver domain, Leu918-Ser1057, yielding the complex AtCRE1-REC-Mg, and a C-terminal truncated receiver domain, Leu918-Phe1048, used for crystallization of the AtCRE1-REC-K complex. The product was inserted into the pMCSG68 vector (Midwest Center for Structural Genomics) and the sequence was confirmed by DNA sequencing. The AtCRE1-REC-Mg protein was overexpressed in *E. coli* BL21 Gold (Agilent) strain, while the truncated AtCRE1-REC-K protein was overexpressed in Magic *E. coli* strain (Midwest Center for Structural Genomics). The cells were cultivated in LB medium with 150 μg/mL ampicillin at 37°C until the optical density (OD) reached 0.8–1. The culture was then cooled down to 20°C and the protein production was induced for 18 h using 0.5 mM of isopropyl β-D-1-thiogalactopyranoside (IPTG). The suspension was centrifuged at ~7, 000 × g at 4°C for 10 min. The pellet was resuspended in cold Binding buffer (50 mM HEPES pH 7.5, 500 mM NaCl, 100 mM KCl, 20 mM imidazole, 1 mM TCEP) and stored at −80°C. The cell pellet was thawed and sonicated for 5 min of probe working time in PULSE mode (4 s on/26 s off) in an ice-water bath. The solution was centrifuged at 27,000 × g for 30 min at 4°C. Then, the supernatant was applied to a column loaded with HisTrap HP Ni-NTA resin (GE Healthcare), connected to VacMan and a vacuum pump. Then, it was washed five times with the binding buffer and eluted using 15 mL Elution buffer (50 mM HEPES pH 7.5, 500 mM NaCl, 100 mM KCl, 400 mM imidazole, 1 mM TCEP). To the eluted sample, a 500 μL aliquot of TEV protease (2 mg/mL) was added to remove His tag. Simultaneously, the mixture was dialyzed overnight at 4°C against dialysis against dialysis buffer (50 mM HEPES pH 7.5, 500 mM NaCl, 100 mM KCl, 1 mM TCEP) using a 10 kDa cutoff Snakeskin dialysis tubing (Thermo Fisher). Next day, the solution was run through the Ni-NTA column again to remove the His-tag and the His-tagged TEV protease. The flow through was collected and concentrated to 2 mL and injected into a Superdex200 16–60 (AtCRE1-REC-K) or Superdex75 16–60 column (AtCRE1-REC-Mg) of an AKTA FPLC system and run using SEC buffer (25 mM HEPES pH 7.5, 50 mM NaCl, 100 mM KCl, 1 mM TCEP).

AtCRE1-REC was analyzed by Native-PAGE (Supplementary Figure 1) in comparison with other proteins, including examples of PR-10 proteins, such as HYP-1 (Michalska et al., 2010), VrPhBP (Ruszkowski et al., 2014), and LlPR-10.2B (Sliwiak et al., 2018). All three exist as monomers and their calculated pI values are similar to that of AtCRE1-REC.

### MS Experiments

The 1st Ni-NTA purification step was similar as above. The eluted sample was concentrated and applied to a Superdex200 16/60 column. The SEC fractions were analyzed by SDS-PAGE and only fractions containing MtCRE1-IC were collected. The sample was divided into two portions. The reference portion was dialyzed against the SEC buffer overnight at 5°C. The second portion was supplemented with ATP and MgCl_2_ at the final concentrations of 0.5 and 2 mM, respectively. The sample was dialyzed against the SEC buffer with 0.5 mM ATP and 2 mM MgCl_2_ at 5°C. The next day, 250 μL of TEV protease (1 mg/mL) were added to each portion, and the dialysis continued overnight. The next day, both portions were applied to Ni-NTA resin to remove the His-tag and MBP fusion (the sample used for SDS-PAGE is named HT2 in Supplementary Figure 5). Thermolysin protease was added to both portions to the final concentration of 0.5 μg/mL. Both samples were dialyzed overnight at 19°C. The next day, each portion was concentrated and applied into a Superdex 75 16/60 column. The eluted fractions were analyzed by SDS-PAGE (Supplementary Figure 5). The samples were further digested with trypsin and analyzed by MALDI and LC/MS on a MALDI-TOF/TOF ultrafleXtreme (Bruker) mass spectrometer; the results are presented in Supplementary Figure 6, Supplementary File 1.

### Crystallization of the Proteins

Purified AtCRE1-REC-K protein was concentrated to 13.3 mg/mL (UV absorption with extinction coefficients of 9970 M^−1^×cm^−1^) and the crystallization experiment was set up in sitting-drop 96-well plates using Index (Hampton Research), BCS (Molecular Dimensions) and Morpheus (Molecular Dimensions) screens. In each drop, 2 μL of the protein solution were mixed with 2 μL of the reservoir solution and the plates were incubated at 19°C. After two days, small needle-like crystals formed in the D2 Index condition containing 0.1 M HEPES pH 7.0 and 30% Jeffamine M-600. AtCRE1-REC-Mg was concentrated to 22.5 mg/mL. MgCl_2_ was added to the protein solution at a final concentration of 2 mM. The same screens were used as above, and further optimization was based on the A4 Morpheus condition containing 0.03 M MgCl_2_, 0.03 M CaCl_2_ in 0.1 M MES/Imidazole at pH 6.5 and 12.5% of each PEG1000, PEG3350 and MPD. The best-diffracting crystal was obtained using the A4 Morpheus condition with 15% MPD.

The solution of MtCRE1-REC after proteolysis was concentrated to 5.5 mg/mL (determined using the Bradford method (Bradford, 1976) because the sequence of the cut fragment was unknown) and subjected to crystallization screening. Crystals grew in A12 Morpheus condition (Gorrec, 2009) containing 0.03 M MgCl_2_, 0.03 M CaCl_2_ in 0.1 M of Tris/BICINE at pH 8.5 and 12.5% of each PEG1000, PEG3350 and 0.1 M MPD.

### X-Ray Data Collection and Processing

X-Ray diffraction data for MtCRE-REC-Ca, AtCRE1-REC-K and AtCRE1-REC-Mg crystals were collected at the EMBL P13 beamline of the PETRA III storage ring at DESY Hamburg, Germany. The raw diffraction data were processed with XDS (Kabsch, 2010). The diffraction data for MtCRE1-REC-Ca and AtCRE1-REC-K were highly anisotropic and were additionally processed with STARANISO (Tickle et al., 2018). Details of data collection and processing are presented in Table 1.

### Structure Solution and Refinement

The crystal structures of AtCRE1-REC-K and MtCRE1-REC-Ca were solved by molecular replacement using PHASER (McCoy et al., 2007) and the coordinates of the AHK5 receiver domain of the PDB model 4EUK. The initial models were built with Phenix.AutoBuild (Terwilliger et al., 2008), after which model corrections were made in several rounds of manual rebuilding in Coot (Emsley et al., 2010) and automatic refinement in Phenix.Refine (Afonine et al., 2012). The initial phases for AtCRE1-REC-Mg were obtained by refinement using the coordinates of the isomorphous AtCRE1-REC-K model.

Three different metal cations were identified at the same site in all three structures. In each structure, the metal was placed according to the electron density, bond lengths, and coordination geometry (Black et al., 1994; Harding, 2002; Yang et al., 2002). The metals were also validated using CheckMyMetal (Zheng et al., 2017).

### Bio-Layer Interferometry

Both MtHPt1 and MtCRE1-IC were purified using similar buffers and protocols as mentioned above. MtHPt1 was run through a SEC column immediately after the first Ni-NTA purification, without His_6_-MBP-tag removal. MtCRE1-IC was purified as described for AtCRE1-REC. The final solution contained MtCRE1-IC free of the MBP fusion.

An Octet K2 (ForteBio, Pall Science) instrument was used for the binding studies. The BLI experiments were performed on Black 96W plates (E&K Scientific). Ni-NTA sensors (ForteBio) were used to immobilize MtHPt1 with the N-terminal His_6_-MBP fusion. The scheme of the BLI measurement was as follows. After 60 s baseline determination in BLI buffer (SEC buffer with 10% Kinetic Buffer (ForteBio, Pall Science), 0.5 mM of ATP and 2 mM of MgCl_2_), the biosensors were loaded for 300 s using a freshly mixed solution of MtHPt1 at 6.3 μg/mL (104 nM) concentration in BLI buffer. A second 60 s baseline in the BLI buffer was recorded, after which the ligand binding was monitored. For the association step, the MtHPt1-loaded biosensors were immersed for 600 s in serial dilutions of MtCRE1-IC at 2.5, 5, 10 and 20 nM concentration in BLI buffer with 0.5 mM of ATP and 2 mM of MgCl_2_. For the dissociation step, the sensors were immersed in BLI buffer for another 200 s. During the entire kinetic assay, the sample plates were kept at 25°C and agitated at 1,000 rpm. Each measurement included a parallel reference, in which the sensors were immersed in the BLI buffer with 0.5 mM of ATP and 2 mM of MgCl_2_ only to monitor unspecific ligand binding to the sensor. Reference-subtracted BLI response curves were used for the determination of the *K*_*d*_ constant and its error. Three replicates were performed for error determination. Inter-step correction and Y-alignment were used to minimize tip-dependent variability. Data were collected and globally fitted by a 1:1 stoichiometry model using the Data Acquisition and Data Analysis Software vHT 11.1 (Forte Bio). The fitting met the quality criteria χ^2^ <3 and R^2^ > 0.96.

## Data Availability Statement

The datasets presented in this study can be found in online repositories. The names of the repository/repositories and accession number(s) can be found below: http://www.wwpdb.org/, 7P8C; http://www.wwpdb.org/, 7P8D; http://www.wwpdb.org/, 7P8E. Raw X-ray diffraction data were deposited in the Macromolecular Xtallography Raw Data Repository (MX-RDR): AtCRE1-REC-Mg, https://doi.org/10.18150/UUYPCU; AtCRE1-REC-K, https://doi.org/10.18150/X9II0M; MtCRE1-REC, https://doi.org/10.18150/DPBWOQ.

## Author Contributions

LT solved and analyzed the crystal structures of AtCRE1 and drafted the manuscript. AU supervised the BLI experiments. MJasi provided physiological background to this work. MJask and MR supervised the work, participated in writing and edited the text. MR solved the structure of MtCRE1. All authors contributed to the article and approved the submitted version.

## Research Involving Plants

Studies complied with local and national regulations for using plants.

## Funding

This project was supported by the National Science Centre grant number: SONATA 2018/31/D/NZ1/03630. MJasi was supported by Polish National Science Centre grant 2015/19/B/NZ9/03548.

## Conflict of Interest

The authors declare that the research was conducted in the absence of any commercial or financial relationships that could be construed as a potential conflict of interest.

## Publisher's Note

All claims expressed in this article are solely those of the authors and do not necessarily represent those of their affiliated organizations, or those of the publisher, the editors and the reviewers. Any product that may be evaluated in this article, or claim that may be made by its manufacturer, is not guaranteed or endorsed by the publisher.
