# Supplementary material for: 3D Domain Swapping Dimerization of the Receiver Domain of Cytokinin Receptor CRE1 From Arabidopsis thaliana and Medicago truncatula
Source: Front Plant Sci. 2021 Sep 24;12:756341. doi: 10.3389/fpls.2021.756341 (PMC8498639; doi:10.3389/fpls.2021.756341)
Supplement: Supplementary file 2 [file Data_Sheet_1.PDF]

Supplementary information

3D domain swapping dimerization of the receiver domain of cytokinin receptor CRE1 from *Arabidopsis thaliana* and *Medicago truncatula*

Linh H. Tran[1], Anna Urbanowicz[1], Michał Jasiński[1,2], Mariusz Jaskolski[1,3], Milosz Ruszkowski[1*]

[1] Institute of Bioorganic Chemistry, Polish Academy of Sciences, Poznan, Poland

[2] Department of Biochemistry and Biotechnology, Poznan University of Life Sciences, Poznan, Poland.

[3] Department of Crystallography, Faculty of Chemistry, A. Mickiewicz University, Poznan, Poland

***Correspondence:**
Milosz Ruszkowski
Institute of Bioorganic Chemistry,
Polish Academy of Sciences,
Noskowskiego 12/14
61-704 Poznan, Poland

E-mail: mruszkowski@ibch.poznan.pl

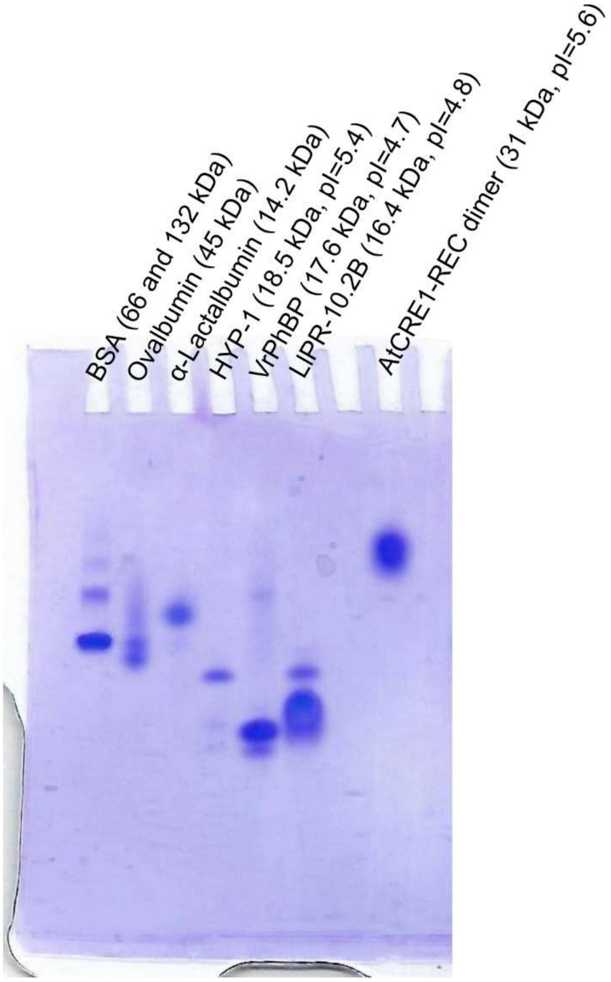

Figure S1: Native polyacrylamide gel electrophoresis (Native-PAGE). Molecular weights and isoelectric points (for proteins of MWs similar to the AtCRE1-REC subunit) are given above the gel lanes. Bovine serum albumin (BSA), ovalbumin, and α-lactalbumin were purchased from Sigma-Aldrich. HYP-1 (Michalska et al., 2010), VrPhBP (Ruszkowski et al., 2014), and LlPR-10.2B (Sliwiak et al., 2018) were produced as referenced. The isoelectric points were calculated using the Expasy ProtParam (Gasteiger et al., 2005) webserver from the protein sequence.

```
AHK5-REC       GSHMETSKPKILLVEDNKINIMVAKSMMKQLGHTMDIANNGVEAITAIN-SSSYDLVLMD    59
MtCRE1-REC     ----LLFGKKILVVDDNVVNRRVAAGALKNFGADVKCADSGKAALEMLQFPHKFDACFMD    56
AtCRE1-REC     ----LLTGKKILVVDDNIVNRRVAAGALKKFGAEVVCAESGQVALGLLQIPHTFDACFMD    56
                        ***:*:** :*  ** . :*::*  :  *:.*  *:  ::   .:*  :**

AHK5-REC       VCMPVLDGLKATRLIRSYEETGNWNAAIEAGVDISTSENEQVCMRPTNRLPIIAMTANTL    119
MtCRE1-REC     IQMPEMDGFEATRRIREMERTANEETNSECGE-----------RKSEFHLPILAMTADVI    105
AtCRE1-REC     IQMPQMDGFEATRQIRMMEKEAKEKTN-----------------LEWHLPILAMTADVI    98
               : ** :**::*** **  *. .: ::                      :***:****:.:

AHK5-REC       AESSEECYANGMDSFISKPVTLQKLRECLQQYLH--------      153
MtCRE1-REC     HATYEECLKCGMDGYVSKPFEEENLYQAVAKFFQTKPTSVDS      147
AtCRE1-REC     HATYEECLKSGMDGYVSKPFEEENLYKSVAKSFKPNPISPSS      140
                 : ***   ***.::***.  ::* :.: : ::
```

Figure S2: Sequences alignment (by webserver Clustal Omega (Madeira et al., 2019)) of three receiver domains of the histidine kinase AHK5, MtCRE1 and AtCRE1. The sequence identity is about 34%. The distance measured in Fig. 4 is between the Cα of Ser892 (beginning of the α5 helix) of AHK5-REC and its corresponding Cα of Tyr965 (in swapping α4 helix) in MtCRE1-REC (boxed).

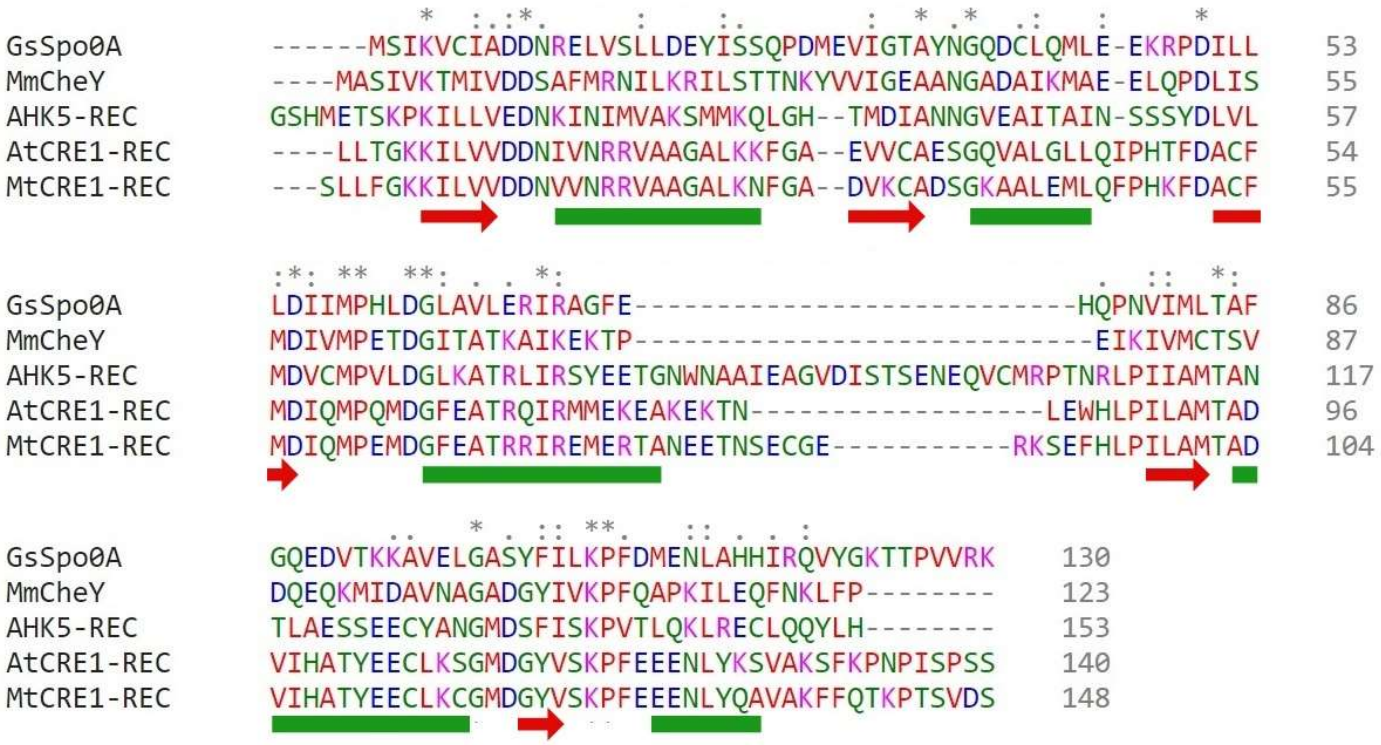

Figure S3: Sequence alignment (by webserver Clustal Omega (Madeira et al., 2019)) of the receiver domain of the response regulator of Spo0A (from *Geobacillus stearothermophilus*) (PDB ID 1QMP) and of CheY (from *Methanococcus maripaludis*) (PDB ID 6EKH) with the receiver domain of cytokinin receptors MtCRE1, AtCRE1 and AHK5. The secondary structure elements are shown as red arrows (β strands) and green bars (α helices).

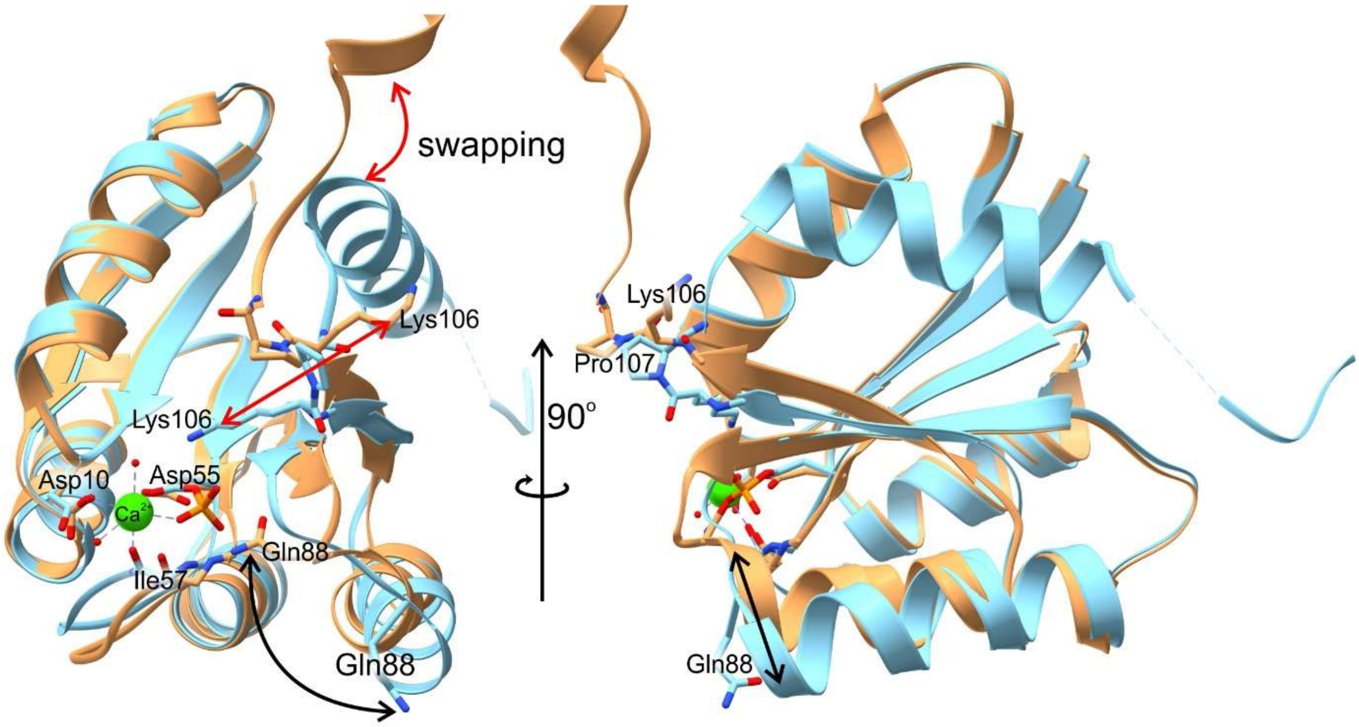

Figure S4: The phosphorylated (blue) and the nonphosphorylated (sandy brown) structure of Spo0A. The 3D domain swapping is triggered by *trans-cis* isomerization of the Lys106-Pro107 peptide bond. It induces the Lys side chain flip of 180° (red arrow) into the metal binding site where it forms a hydrogen bond with oxygen atom of the phosphate. However, it is also noticeable that the α4 helix undergoes some displacement upon the phosphorylation event (black arrow).

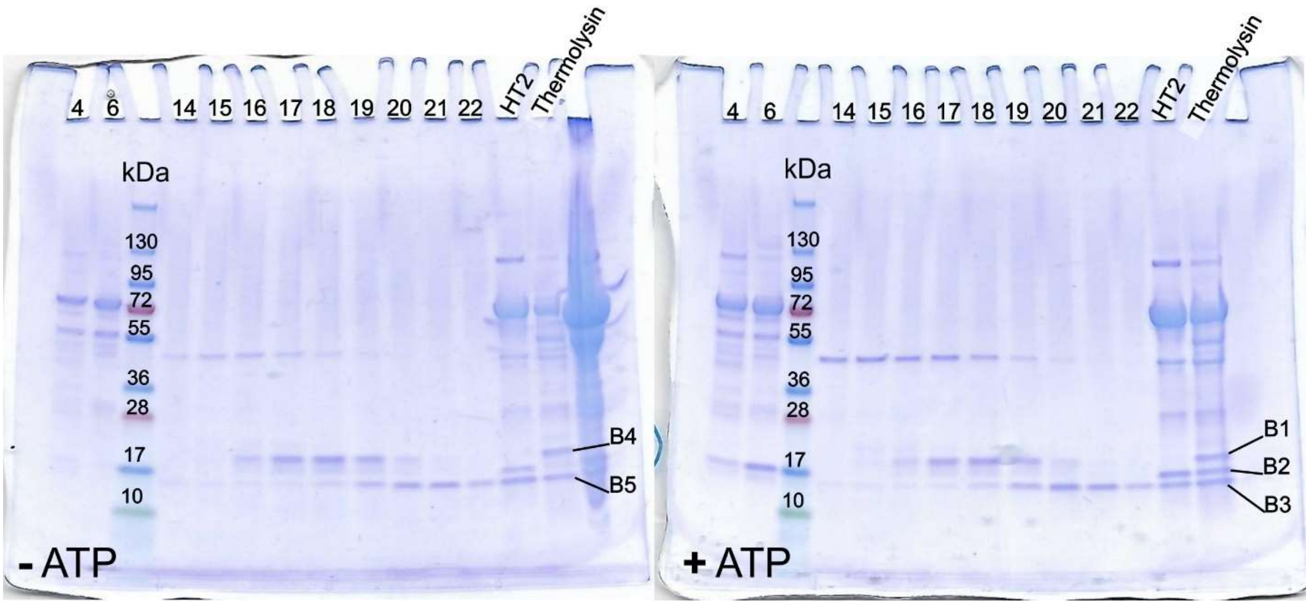

Figure S5: SDS-PAGE gel of the MtCRE1-IC protein solution after processing with thermolysin, followed by size exclusion chromatography. “HT2” is the solution after removal of the MBP fusion and His-tag. “Thermolysin” is the solution after overnight incubation with thermolysin, before SEC. The lane numbers correspond to the eluted fractions in SEC. A new band (B2) appears in the lane “thermolysin” in the solution incubated with ATP. The bands from B1 to B5 were cut and sent for MS analysis.

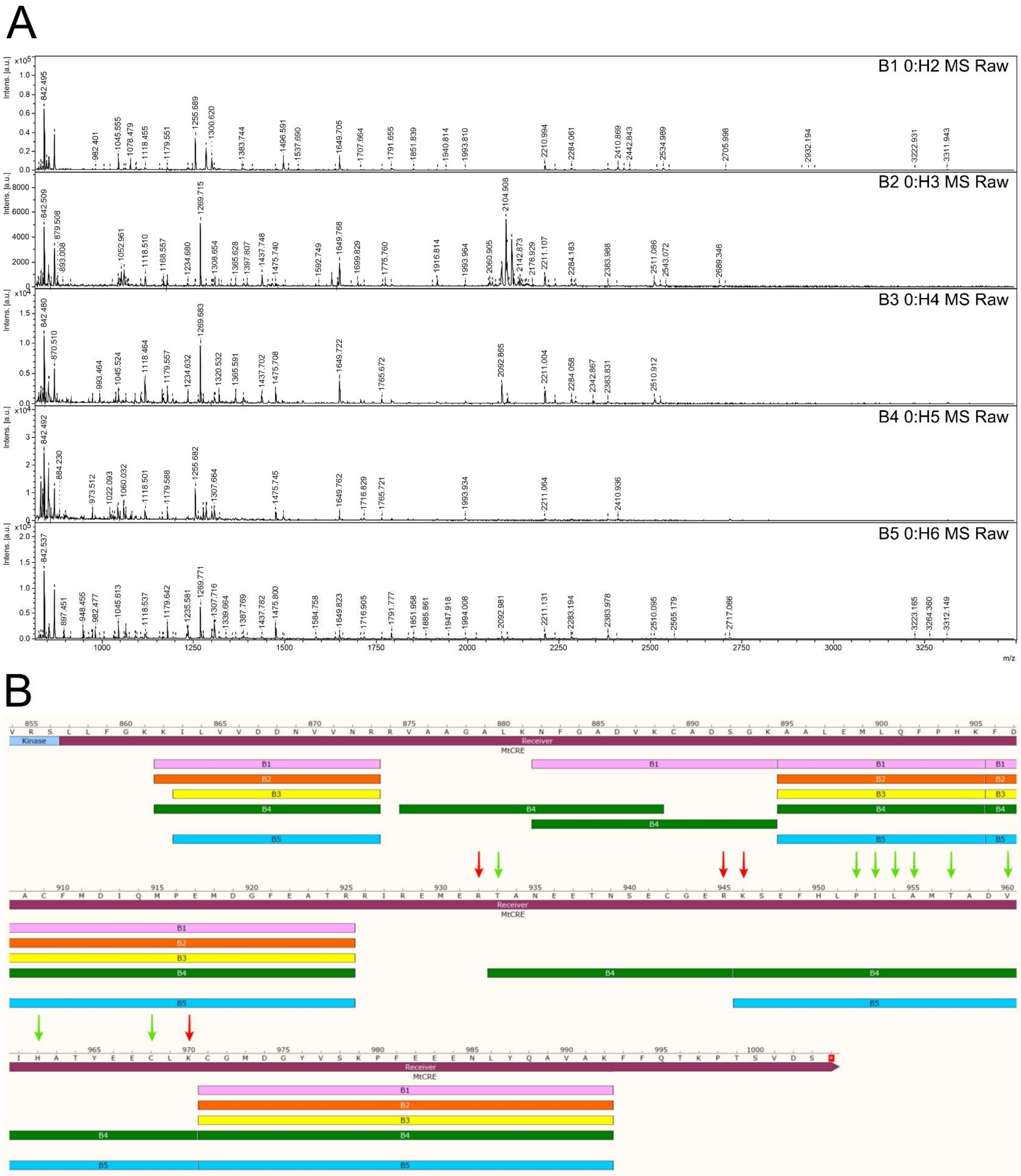

Figure S6: Mass spectrometry results. The samples were cut from the corresponding bands (B1-B5) of the SDS-PAGE gel in Fig. S5 and were further digested with trypsin. Raw results are presented in panel A, while panel B shows the sequenced peptide map for MtCRE1-REC domain. In the supplementary file S7, peptides corresponding to MtCRE1-REC domain fragments are

highlighted using the same colors as in panel B. The digestion patterns are similar except for the fragment 933-970 corresponding to the protruding helix α4 (951-970) and strand β4 (953-956) in the sample with (B1, B2, B3) and without (B4, B5) ATP. The green and red arrows indicate the cleavage sites of thermolysin and trypsin, respectively, predicted by the PeptideCutter webserver (Gasteiger et al., 2005).

References

Gasteiger, E., Hoogland, C., Gattiker, A., Duvaud, S.E., Wilkins, M.R., Appel, R.D., and Bairoch, A. (2005). "Protein Identification and Analysis Tools on the ExPASy Server," in *The Proteomics Protocols Handbook,* ed. J.M. Walker. Humana Press), 571-607.

Madeira, F., Park, Y.M., Lee, J., Buso, N., Gur, T., Madhusoodanan, N., Basutkar, P., Tivey, A.R.N., Potter, S.C., Finn, R.D., and Lopez, R. (2019). The EMBL-EBI search and sequence analysis tools APIs in 2019. *Nucleic Acids Res* 47**,** W636-W641. doi: 10.1093/nar/gkz268

Michalska, K., Fernandes, H., Sikorski, M., and Jaskolski, M. (2010). Crystal structure of Hyp-1, a St. John's wort protein implicated in the biosynthesis of hypericin. *J Struct Biol* 169**,** 161-171. doi: 10.1016/j.jsb.2009.10.008

Ruszkowski, M., Sliwiak, J., Ciesielska, A., Barciszewski, J., Sikorski, M., and Jaskolski, M. (2014). Specific binding of gibberellic acid by cytokinin-specific binding proteins: a new aspect of plant hormone-binding proteins with the PR-10 fold. *Acta Crystallogr D Biol Crystallogr* 70**,** 2032-2041. doi: 10.1107/S1399004714010578

Sliwiak, J., Sikorski, M., and Jaskolski, M. (2018). PR-10 proteins as potential mediators of melatonin-cytokinin cross-talk in plants: crystallographic studies of LlPR-10.2B isoform from yellow lupine. *FEBS J* 285**,** 1907-1922. doi: 10.1111/febs.14455
